# Secondary childhood glaucoma in neurofibromatosis type 1: an unusual corneal leukoma case report

**DOI:** 10.3389/fonc.2024.1469969

**Published:** 2024-11-28

**Authors:** Ling Ying Ge, Xin Tian, Han Mu Guo, Xue Yin

**Affiliations:** Department of Ophthalmology, the First Affiliated Hospital of Soochow University, Suzhou, Jiangsu, China

**Keywords:** neurofibromatosis type 1, corneal leukoma, atrial angle dysplasia, secondary glaucoma, children

## Abstract

Neurofibromatosis type 1 (NF1) is a rare autosomal dominant disorder that affects the skin, eyes and peripheral nervous system. It is rarely associated with glaucoma, especially in pediatric patients. We herein report an unusual case of corneal degeneration in a child with NF1, characterized by peripheral corneal leukoma and a membrane under Descemet’s membrane. This finding offers new insights for the ophthalmic diagnosis of NF1.

## 1 Introduction

Neurofibromatosis is a heterogeneous group of hereditary cancer syndromes that lead to tumors of the central and peripheral nervous systems, as well as other organ systems. By far the most common form is neurofibromatosis type 1 (NF1, 96%), followed by neurofibromatosis type 2 (NF2, 3%), and schwannomatosis. The clinical manifestations of patients with NF1 are diverse and the typical clinical manifestations are cafe-au-lait spots, freckling in the axilla and groin,lisch nodules, and neurofibromas (1). Type 1 neurofibromatosis (NF1)-associated glaucoma is a rare manifestation, occurring in approximately 1%-2% of NF1 patients (2). In the case, the child exhibited corneal degeneration, which was an aspect not previously reported in relation to NF1.

## 2 Case description

A 9-year-old girl presented to our ophthalmology department on December 2023. She was the first child, born of non-consanguineous marriage with an uneventful natal and perinatal history. She was noticed to have intermittent left eye redness and protrusion since six months. She was found to have reduced left eye vision in another ophthalmological facility. She was managed for glaucoma with eye drops, but no detailed eye examination report was provided upon presentation.

**Figure 1:**
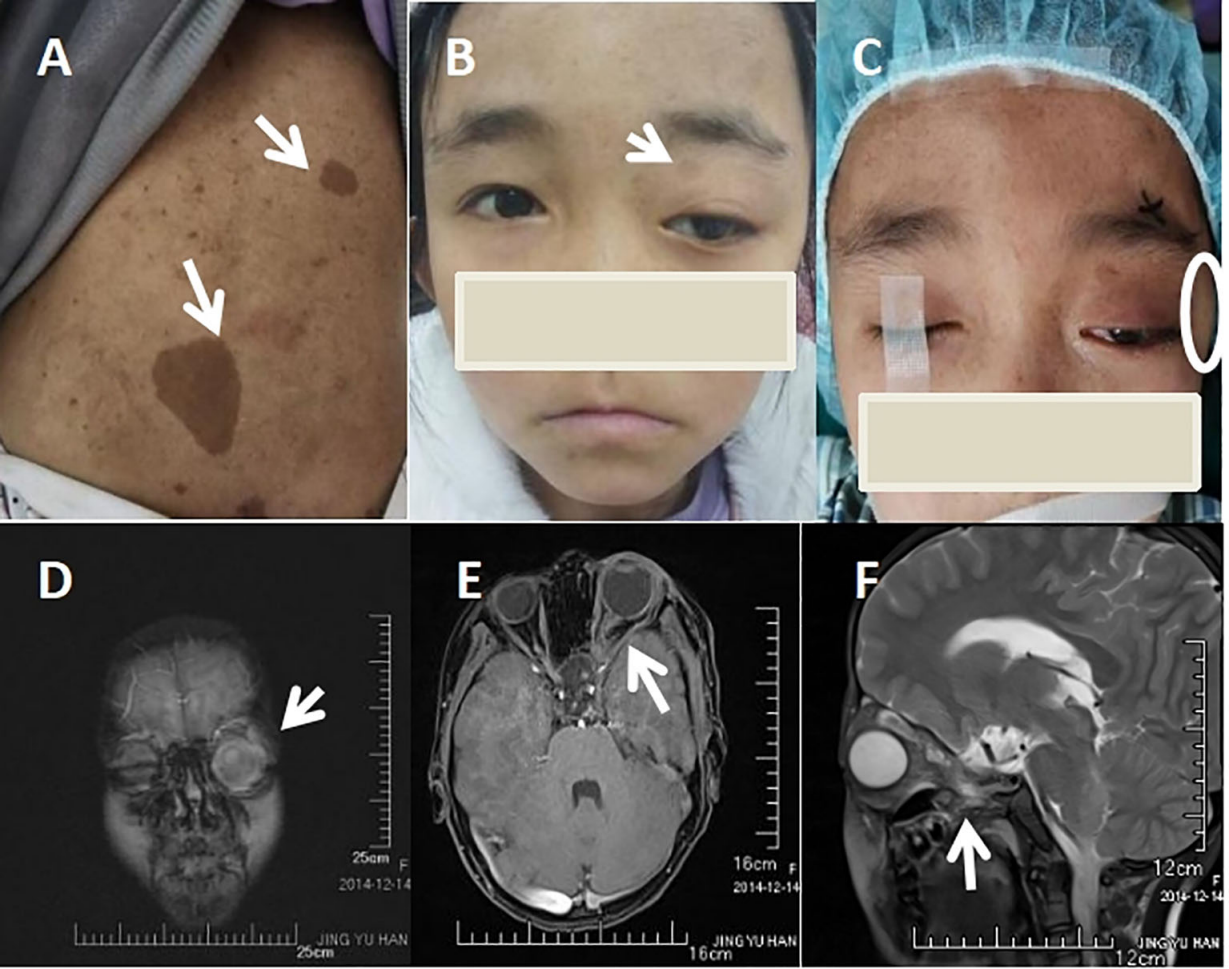
Skin and Imaging Features. **(A)** Multiple café-au-lait spots in back (white arrow heads). **(B)** Frontal picture of the patient showing proptosis in the left eye(white arrow heads). **(C)** External Appearance of the patient’s left orbital space occupying lesion(white circle). **(D)** Orbital space occupying lesion of left eye(white arrow heads). **(E)** Left optic nerve tortuosity and thickening of both the nerve and its sheath(white arrow heads). **(F)** Lateral view of optic nerve tortuosity (white arrow heads).

**Figure 2:**
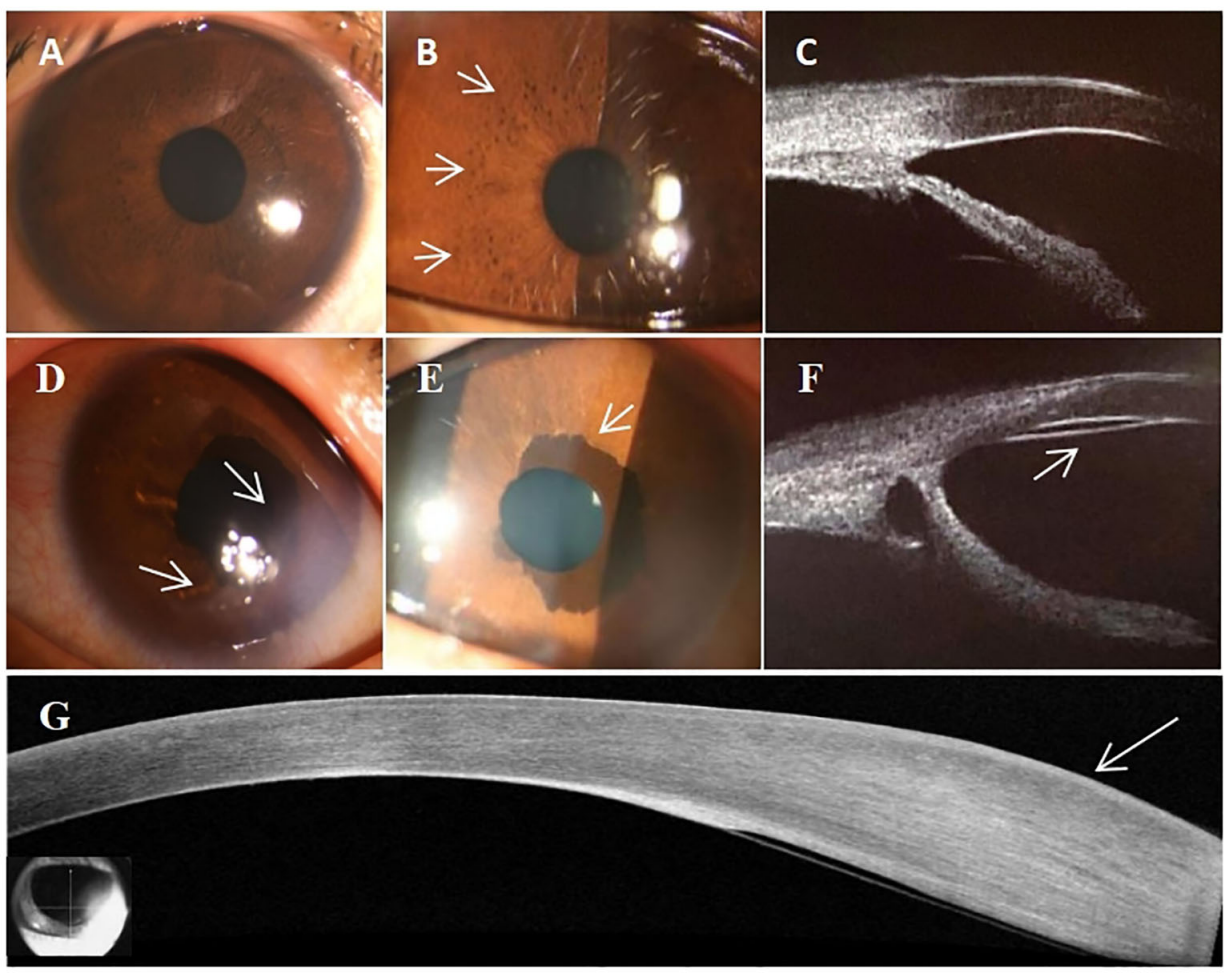
Characteristics of Ocular Manifestations. **(A)** Slit-lamp biomicroscopy picture of right eye. **(B)** Right eye showing lisch nodules (white arrow heads). **(C)** Right eye showing an open anterior angle without abnormalities. **(D)** Peripheral corneal degeneration in the left eye (white arrow heads). **(E)** Ectropion uveae in the left eye (white arrow heads). **(F)** Abnormal anterior angle with the presence of a continuous membrane layer in the posterior elastic lamina (white arrow heads). **(G)** The OCT of abnormal thickening of the posterior elastic lamina and the presence of a continuous membrane(white arrow heads).

Her physical examination showed no dysmorphic features, but multiple café-au-lait spots on her back (**Figure 1A**), along with proptosis of the left eye (**Figure 1B**) and an orbital space occupying lesion (**Figure 1C**). The ocular evaluation indicated a best-corrected visual acuity of 20/40 in the right eye and 20/80 in the left eye. Intraocular pressure (IOP) readings were 17 and 22 mmHg in the right and left eye. Slit-lamp biomicroscopy showed multiple lisch nodules on the surface of the iris in the right eye (**Figures 2A, B**) and leukoma in the peripheral portion of the left eye (**Figure 2D**), along with ectropion uveae (**Figure 2E**). Clinical specular microscope showed decreased endothelial cell density and increased polymegathism in the left eye. Her visual field showed glaucoma progression in the left eye and eyeground photography disclosed cup-to-disc ratios of 0.6 in the left eye, respectively(right eye axis: 18.4 mm, left eye axis: 22.1 mm).

Ultrasound biomicroscopy identified a normal open anterior angle in the right eye (**Figure 2C**), however, in the left eye, the anterior angle was abnormally developed and closed in partial quadrants, with the presence of a continuous membrane layer after the Descemet’s membrane (**Figures 2F, G**). Further optical coherence tomography (OCT) of the anterior segment indicated localized corneal edema with leukoma, abnormal thickening of the corneal stromal layer, and a continuous membrane (**Figure 2H**).

Orbital magnetic resonance imaging (MRI) revealed the patient’s left intraorbital lesion with thickened optic nerve tortuosity (**Figures 1D–F**). The child had no familial history of glaucoma but the familial history of NF1 from her father which was confirmed through genetic examination. Our patient was assessed by pediatric dermatology, neurology, and genetics and was clinically diagnosed as NF1. NF1- associated glaucoma was our working diagnoses.

Owing to poor visual outcome in the left eye, no further intervention other than continuing maximal medical therapy of Brinzolamide + Tafluprost eye drops in left eye was suggested. A month subsequent to the initial visit, IOP was maintained in the range of 7-16 mmHg. The importance of continued ophthalmologic and neurological monitoring was stressed to the family.

## 3 Discussion

Neurofibromatosis is an autosomal dominant genetic disorder that can cause damage to multiple systems (3). Glaucoma is a rare manifestation of NF1, especially in children, and the ophthalmic literature regarding glaucoma and NF1 indicates that NF1-associated glaucoma is classified as secondary childhood glaucoma (4–6). NF1-associated glaucoma is typically unilateral and diagnosed from birth to 3 years, although diagnosis in childhood and adolescence have been described. NF1 can manifest in various ophthalmic presentations, including lisch nodules, optic gliomas, and neurofibromas of the eyelid, conjunctiva, and orbit (7–9). The underlying mechanism of NF1-associated glaucoma is multifactorial, with potential causes including (1) direct infiltration of the anterior chamber angle by neurofibromas, (2) secondary angle closure resulting from neurofibromatous thickening of the ciliary body and choroid, (3) fibrovascularization leading to synechial angle closure and neovascular glaucoma, and (4) developmental angle abnormalities (10–12).

In this case, the diagnosis of NF1 was confirmed through genetic examination which showed mutations in the gene NF1, aligned with some clinical manifestations including cafe-au-lait spots on the back, lisch nodules in the right eye and ectropion uveae in the left eye. Secondly, based on the patient’s ocular manifestations including proptosis of the left eye, the high IOP in the left eye, the growth of left eye axis, along with glaucomatous visual field defect and cup-to-disc ratios of 0.6 in the left eye, we further confirmed the diagnosis of NF1 secondary glaucoma. With the manifestation of Ultrasound biomicroscopy identifying the anterior angle of the left eye which was abnormally developed and closed in partial quadrants, we speculated that the disease’s likely cause is the abnormal development of the anterior chamber induced by neurofibroma.

In addition, orbital magnetic resonance imaging (MRI) of the child revealed left optic nerve tortuosity and thickening of both the nerve and its sheath. Several studies have demonstrated that optic nerve tortuosity is linked to the development of optic glioma in patients with NF-1 (13). Therefore, we can speculate that the patient is highly likely to have NF1-associated optic nerve glioma.

The precise mechanism by which this patient developed specific corneal leukoma remains unknown, and similar ocular manifestations of NF1 in pediatric patients have not been documented in previous studies (8). The patient’s corneal leukoma appearance differ from Haab’s striae observed in primary open-angle glaucoma (POAG) (14, 15). Haab’s striae usually appear as curved horizontal fractures in the posterior elastic layer, indicating a thickened edge of the posterior elastic layer that protrudes into the anterior chamber with a knob-like structure. The characteristic feature of the corneal leukoma in our case is the extensive thickening of the corneal stromal layer, with no rupture of the Descemet’s membrane, presenting a continuous membrane (15).

The possible mechanism of the corneal leukoma may relate to the instability of endothelial cell in corneal endothelium, which disturbs the balance of stromal hydration, leading to changes in corneal transparency and reduced corneal sensitivity (16–18). As reported, the functional inactivation of the NF1 gene leads to activation of the MAPK pathway which results in cellular proliferation of many neural-crest derived cells and development of neurofibromas and pigmentary abnormalities in NF1 (19, 20). As corneal endothelium is embryologically derived from the neural crest (18), it is possible that activation of the RASRAF-ERK-MAPK pathway in corneal endothelial cells may trigger endothelial cell proliferation similar to in other neural crest-derived cell populations in NF-1. Moreover, some reports indicate that patients with NF1 exhibit abnormal proliferation of corneal endothelial cells, increase of corneal endothelial cell density, variation in corneal endothelial cell size, and a higher incidence of corneal polymegathism and pleomorphism (changes in cell shape) (21). The reports suggest that coefficient of variation of cell area (CV) reflects the functional reserve of endothelial cells and is a highly sensitive indicator of early endothelial instability (16, 18). As a result of CV increase in NF1 patients, the functional reserve of the endothelium reaches its limit, corneal endothelial function is compromised, disrupting the hydration balance of the stroma and leading to corneal edema and opacification.

However, as our patient’s age being only 9 years and the conservative treatment, the eye specimen and pathological samples of this patient could not be available to our case report for further research of the mechanism. Additionally, due to this being a single case with a short follow-up duration, the relationship between corneal opacity and NF1-associated glaucoma needs further study to prove.

As a result of the limitations mentioned, we additionally suggested the pathological mechanisms from her genetic examination. In the genetic examination, the suspected variant genes of this child associated with corneal opacities include SLC4A11 and PLG. The mutations in SLC4A11, which relate to diseases of the corneal endothelium (22–24), manifest progressive corneal edema and opacification with a bluish-gray ground glass appearance and the incrassation of the cornea. And the plasminogen protein encoded by PLG gene is a serine protease that circulates in blood plasma as an inactive zymogen and is converted to the active protease, plasmin, by several plasminogen activators (25, 26). Plasmin activity is upregulated in normal corneal wound repair, leading to the continued deposition of fibrin in corneas and thickening of the corneal stroma which may directly or indirectly cause the persistent corneal opacification.

In conclusion, we present the first report of corneal leukoma manifestation in pediatric patients, providing a novel indication of ocular manifestations in NF-1. We propose that corneal opacity in pediatric NF1-associated glaucoma may be attributed to damage of the corneal endothelial cells, potentially indicating abnormalities in the development of anterior chamber angles and thus suggesting a predisposition to glaucoma. For the rarity of the NF1-associated corneal opacity being studied, further researching the mechanistic pathways of corneal opacity in NF1 patients will be a challenging and long-term project.

## Data Availability

The datasets presented in this article are not readily available because of ethical and privacy restrictions. Requests to access the datasets should be directed to the corresponding author.
